# How Flow Experience and Self-Efficacy Define Students’ Online Learning Intentions: View From Task Technology Fit (Framework)

**DOI:** 10.3389/fpsyg.2022.835328

**Published:** 2022-03-15

**Authors:** Hai Huang, Yong Wang

**Affiliations:** ^1^School of Public Affairs, University of Science and Technology of China, Hefei, China; ^2^Educational Training Center, Changzhou Liu Guojun Vocational Technology College, Changzhou, China; ^3^School of Information Science and Technology, University of Science and Technology of China, Hefei, China

**Keywords:** task-technology fit, business simulation games, gamification, flow experience, interactivity, online learning, online learning technology, self-efficacy

## Abstract

The ongoing pandemic has transformed communication modes globally. Especially in the case of higher education, where countermeasures against coronavirus disease 2019 (COVID-19) have affected students’ learning experience. This study emphasized the case of business simulation games, where critical factors were underlined to define learners’ intention to use an online learning environment through the lens of task technology fit (TTF) as a theoretical stance. This study considered the statistical analysis of 523 students who attended the business simulation module online at the tertiary level of education. Findings conclude that flow experience is the most critical factor to define learners’ perceived TTF in the case of an online learning experience. However, the learners’ self-efficacy is significant enough to map learners’ intentions to use an online environment for learning. The study discussed several theoretical and practical implications for learners’ educators and policymakers.

## Introduction

Business simulation games (BSGs) have been used to bridge the gulf between practice and theory in scientific studies (Buil, 2018). These games reproduce various aspects of the actual environments through the simulated environment (Faria et al., 2009). Subsequently, BSG encompasses a vital learning tool to engage students by improving their cognition, analytical abilities, and decision-making skills. BSG has been employed at a higher pace within academia to harness the real potential of students (Clarke, 2009). BSG has put comprehensive and wide-ranging verities in education and training that enable students to learn and experience the scientific environment (Merkuryev and Bikovska, 2012). The literature has indicated that BSG has various advantages, such as increasing learning, knowledge, and skills. For instance, it enables students to learn scientific knowledge through games with the best academic performance (Levant et al., 2016). Along with scientific learning, many other skills are formed, evolved, and developed by BSG in students, such as general competency, reduced uncertainty, decision-making, and analytical skills (Hernández-lara et al., 2018).

Improved communication skills, problem-solving, teamwork, and adaptability to new conditions have also been spurred from the interaction and learning of respective games (Mammadova, 2021). Correspondingly, it adds to the impressive academic performance of students because they acquire skills to produce scientific models and manage them (Pedrosa-ortega et al., 2021). In the context of gamification and design, the simulated environment can be captioned as a strategic effort to enhance the organization, services, system, and activities to acquire real experiences (Bitrián et al., 2020). Gamification has been widely accepted in education with the belief stemming from the potential to create engagement, behavioral changes, motivation, competition, collaboration, and performance (Dichev and Dicheva, 2017). The utility of gamification in education is embedded in the fact that students learn with higher efficiency and interest when they are having fun. It also entails peer competition, point scoring, learning assistance, and assimilation of new information to check their knowledge. Motivation has been a challenging factor in education; thus, gamification is significant because it sustains students’ motivation in learning (Li et al., 2013).

This study aims to underline the following two research questions. RQ1: How interactivity and flow experience help to define students’ perceived task technology fit (TTF) in the case of an online learning environment (a case of BSG) (Dirsehan and Cankat, 2021). RQ2: How scientific (inquiry-based) self-efficacy can increase the adaption of an online learning environment (a case of BSG) (Cen et al., 2020). The study intends to contribute to the existing literature by highlighting the strategic role of real-time interactivity and active control to define the meaningfulness of technical characteristics (Ferreira et al., 2021). In the previous pool of literature addressing TTF (Cooper et al., 2021), research initiatives usually caption “technology and task characteristics” as exogenous factors to map TTF. However, this research initiative extends the detail of technology on the basis of interactivity, flow, and quality of graphical design. It is the novelty of the study that TTF is taken deep into the broader spectrum of the study. In the existing pool of literature, TTF has only been investigated by the same construct. However, in the present context of the study, TTF has been measured in the context of learning only, and its construct has been prepared through underlined variables of the study. Furthermore, task-related efficacy mapped by computing scientific (inquiry-based) self-efficacy (Zulfiqar et al., 2019) among higher education level students in the online setting is the novel contribution of the study.

By having insights into the existing pool of literature about business simulation, this study takes a deep overview of the origin, development, and evolution. It also entails the definitional framework and the varying definition of business simulation games. Furthermore, this study has been laid on TTF and frames the hypothesis on the basis of the postulates remarked by TTF. It also differentiates measurements of TTF at the organizational level and explains why it has been measured at the individual level in the present context. The study proposes the hypothesis of flow experience, real-time interactivity, and active control.

## Business Simulation Games (Literature Review)

Online business games have emerged as the alternative and effective means of learning globally. With innovation, creativity, and improved learning, they are meant to change the landscape of education (Hernández-Lara et al., 2019). For educators, games must not be necessarily funny but engaging, concentrating, and leading to implicit or explicit learning (Kamath et al., 2017). Despite their commercial purpose, many games are present in the market, but no uniform pedagogy has been developed (Giraud-Carrier et al., 2021). Nonetheless, literature on BSG and relationships with the particular types of learning are scarce (Young et al., 2012). The origin of games in learning can be traced back to two approaches. Some found the basis in the behaviorist model, whereas others tended to experiential, socio-cultural, and situated pedagogical methods. The primary purpose of designing and incorporating BSG into education depends on relevant pedagogy and essential mechanism (Guillén-Nieto and Aleson-Carbonell, 2012). Some of the serious games are structured to remedy mental ailments, such as attention deficit in children. These games are the source of training and make children focused and attentive. Accordingly, BSG can be used in problem recognition and solving, self-monitoring, long, short-term memory, decision making, and increased social skills, such as negotiation and collaboration (Eabrasu, 2021). BSG fosters practical approaches to learning and students’ skills before they enter the real world. Through these games, students can select actions and encounter experiences and certain outcomes of that action (Stummer, 2021).

The origin of the BSG can be traced back to the 1950s when games were designed in the military war games. Business associations, industrial firms, and educational institutes have designed several business games in the following decades. Consequently, the University of Washington has been using BSG in classrooms since 1957. Perhaps BSG has been used as an educational tool for the past 50 years and has gained an important place from the supplemental exercise to the key method of business instructions (Humpherys et al., 2021). Greenlaw and Wyman, 1973. captioned BSG an exercise based on the outcomes of decision-making revolving around some business models, in which individuals experience the reality by supposing specific roles present in the game. Furthermore, literature has remarked that the management game is a simulated experience of the situation, which comprises the delusion of the reality in which one can experience the consequences by participating in it, specifically in the context of business and economics.

Contrarily, Faria et al. (2009) defined BSG from an educational perspective and refer to it as an outcome from education technologists to digital natives, the new generation who have been outstretched on interactive games and digital tools. Perhaps this generation comprehends BSG as a part of the natural process to encounter education through games. BSG has been employed within various educational domains, but its core purpose remains to be training. Elion and Greenlaw first categorized the games by their design characteristics (i.e., computer or non-computer, interacting or non-interacting, and functional or non-functional as per their functional application); they provided instructions for new methods for selling and general management programs, established an investigation on the decision-making process, and at last examined individual behavior in the team (Repository and Centre, 2021).

## Theoretical Framework and Hypotheses Development

A surge to map out the TTF theory in the spectrum of Information Systems (ISs) literature is notable (Wang et al., 2021). The theory has been used at the organizational level; meanwhile, its potential implications have been applied at the individual level (Goodhue and Thompson, 1995). The basis of TTF sprawls from organizational contingency theory by establishing the premise that different technology characteristics significantly impact the performance concerning the task and various situational factors. Eventually, TTF has been used to quantify the beneficial role of technology within a system to evaluate the association between technology and the tasks that technology seeks to accomplish (Rai and Selnes, 2019).

The fundamental postulate of the TTF narrates that consequences or results are based on the alignment or extent of fit between the information and the executed task. Eventually, IT significantly affects the individual’s performance and matches the particular task to be performed (Alyoussef, 2021). Goodhue and Thompson (Park, 2019) formulated the measure of TTF, which entails eight features: quality, authorization, locatability, ease of use, compatibility, systems reliability, production timelines, and relationship with the users. Nevertheless, TTF has been a vital predictor for enhancing any performance and effectivity. The primary purpose of the model by Goodhue and Thompson (1995) has been at the individual level. Seebacher et al. (2020) put forward the extension of this model at the group level. Various modifications have been done in this theory accordingly. However, the theory has been mapped out at the individual level irrespective of its few limitations. TTF is the correspondence between the individual abilities, tasks requirements, and the extent of functional aspects of the technology. In case the gap increases between tasks needs and the functionality of technology, TTF is reduced. TTF increases when a match between these two vital factors happens. The utilization of technology relies on individual behavior and a particular social setting. The frequency of technological use and the diversity of particular applications have a significant role in determining individual performance. Consequently, belief toward technological usage, outcomes of technology use, and social norms influence the individual to use technology or not (Furneaux, 2012).

Task technology fit digs out that technology is apprehended as a tool by individuals to accomplish their particular tasks. Somehow, in the context of this model of the study, technology refers to BSG, different software, and support services that are meant to provide ease in the learning process within the academic circle (Al-rahmi et al., 2020). The model intends to carve out the more specific effects of BSG on the students’ academic performance. Furthermore, in the underlying mapped out model, graphical attraction, network/connectivity support, self-explanatory content, and interaction have been given basis as a technology characteristic.

### Graphical Attraction

The graphical attraction has been a determining factor in academic performance because it increases students’ exposure and confidence. Graphs become vicarious to understand particular scientific concepts. Seebacher et al. (2020) employed the graphical multiuser environment (hereafter as MUVE) to involve pupils in science inquiry learning and investigated self-efficacy. The study found that MUVE has been appealing and surges students’ science learning. Furthermore, the self-efficacy of students exposed to MUVE was higher than the control group. Graphical attraction is subject to creative thinking and novelty. The visual presentation in computer games forges the new culture of learning that corresponds to the interests and habits of the students (Mercan and Tuzun, 2020). It can bridge students’ learning and the problem-solving approach to accelerate their academic performance (Giusti et al., 2021). The rationale is embedded in the fact that games offer pupils the initial problems through graphs and provides the opportunity to seek out their problems on their own. Having graphical attraction, the students’ concentration increases as they observe, explore, manipulate, test, pose new questions, and predict the phenomenon by experiencing it through the virtual world. The graphs make science or any other topic a method, process, and quest for knowledge. Such kind of virtual participation of students enabled an environment and shared norms where individuals practice science through their common or routine language (Rai and Selnes, 2019). Thus, this study provides the following framed hypothesis:

H1: A higher degree of graphical attraction increases the perceived TTF among online learners (a case of BSG).

### Real-Time Interactivity

Real-time interactivity is captioned as the users’ perception or the social indication regarding the connection. Interactivity is a much-prioritized matter for companies. Anthony et al. (2021) articulated the significance of commitment within the spectrum of virtual social circumference. It has replaced traditional dimensions in different fields of the world. Perhaps critical socio-technical phenomena that are not comprehended by users efficiently have arisen. Three dimensions have characterized real-time interactivity: active control, two-way communication, and synchronicity, which is elucidated as the extent to which users become involved in the communication and the simultaneous response they receive (Mensah et al., 2021). Eventually, this synchronicity or the third dimension is known as real-time interactivity. Etemad-sajadi (2016) considered the real-time interactivity dimension and put it as the degree to which customers can participate in altering the content and form of the respective environment in real-time. The central theme of the concerned author revolves around the interaction of the individual and the environment. The interest of participants’ interest escalates when they encounter cocreation, unique experiences, and more involvement in the real-time environment (Hong, 2021). The higher usage of BSG influences students’ capabilities to grasp fundamentals and critical consents, enhancing their academic performance. The following hypothesis has been carved out on the basis of the aforementioned discussion.

H2: The higher degree of real-time interactivity increases the perceived TTF among online learners in the case of BSG adoption.

### Flow Experience

Flow refers to individuals’ overall sensation when they execute an action with their optimum output. It is the individual experience to exert their best effort. In case of the high state of flow, users indulge in their respective activities. They become unaware of their surrounded environment by elapsing time at a higher pace (Zou et al., 2021). Individuals extract pleasure from the flow experience. Keeping in view the vital importance of the flow experience, it has a high breadth of literature in the IS domain. Flow experience has been employed in the context of augmented reality to dig out its impact on individual behavior (Brannon et al., 2021), online games (Carlo et al., 2021), and social media (Xu et al., 2021). Furthermore, students develop a state of flow during studies through BSG; subsequently, they seek greater pleasure, which leads to better academic performance. Conversely, poor flow experience in BSG offers individuals poor services and products (Au, 2020). The good experiences of learning have been positively associated with the flow in terms of BSG. Thus, a mental state arises in students, where they are fully focused and energized in their scientific learnings. Students are pleasurably absorbed within BSG, which positively affects their performances (Central, 2017). The rationale behind this fact lies in the motivation and the engagement that games produce for the students. It has been inferred that flow experience within the context of SG fosters students’ perceived learning, generic skills, and satisfaction (Torre et al., 2021). Thus, the following hypothesis is formulated:

H3: The media enriched flow experience increases the perceived TTF among online learners in the case of BSG adoption.

### Active Control

Active control is defined as an individual’s ability to participate voluntarily and technically exert an impact on communication. This communication is a two-way communication between users and the messages on the Internet and between users and other users (Etemad-sajadi, 2016). Active control with reference to learning have been found with promising outcomes because it affects cognitive learning skills (Sivakumar, 2016). Studies have reflected that active control with reference to BSG and the academic perspective operates as the tool in which the signal fostered by the movement from the control column is entered within the system; thus, it integrates different parameters and produces the output signal to the control in the particular game (Wang et al., 2021). In the scientific learning, intuitive sensory is associated with finger and hand movements. It provides the individual with the optimal awareness and attachment to reinforce their performance. The active control within BSG provides ease and puts forward physical feelings to its users, which engage them and improve their academic performance (Au, 2020). Consequently, the active control environment becomes a motivating factor for the students to complete their actions through BSG. Thus, the following hypothesis is formulated.

H4: The higher degree of active control increases the perceived TTF among online learners in the case of BSG adoption.

### Task Technology Fit (Business Simulation Games)

Business simulation games have inferred TTF in the context of the current mapped out model. It has been designed as a mediator with reference to different variables leading toward academic performance. BSG has attained concertation by educators, managers, and practitioners in recent years. They are categorized as education games, digital game-based learning, serious games, and applied games (Zulfiqar et al., 2019). The simulations are models that reflect critical real-world systems. They forge the models’ mental models in the students because their basic purpose is to boost the students to perform better. The features of BSG match the academic task that students perform in their studies; thus, their scientific learning is improved. BSG has a unique feature of the graphical presentation that plays a significant role in the users’ satisfaction to gain the maximum output (Lovin et al., 2021). Graphical attraction is a psychological condition that alters individual interest and increases concentration. Real-time interactivity and BSG rely on the particular structure of the interactivity that entails communication and some of the critical activities framed by the students, such as engaging, questioning, reflecting, elaborating, problem-solving, discussing, and analyzing (Ackerman and Gross, 2021).

As far as the relationship between the flow and the BSG is concerned, the existence indicates that flow has been an influential construct to determine the relationship between human and computer interactions (Bolotaolo et al., 2021). Furthermore, the flow has also been examined within the online environment to predict user intentions (Rodríguez-ardura and Meseguer-artola, 2019). According to Pai and Yeh (2014), social constructivism perceives knowledge constructed between people by a social process of interacting. The relationship between interactivity and learning outcomes depends on the nature of the interactivity, which involves communication and other complex activities developed by the learners, such as engaging, reflecting, questioning, answering, elaborating, discussing, problem-solving, constructing, and constructing analyzing.

Active control has an interesting association with BSG in academic performance. Active control spurs students’ engagement by providing them with fun and entertainment. It led toward knowledge construction and escalated academic performance (Ruggeri et al., 2019). Nevertheless, based on the earlier discussion, the following hypothesis has been made to seek the mediating role of self-efficacy over the relationship between perceived TTF and intentions to use the online setting to learn business simulation.

H5: The higher degree of perceived TTF increases online learners’ intentions to use the digital environment (as a case of BSG).

### Scientific Inquiry-Based Self-Efficacy

Scientific inquiry has been much debated among the academic circles in the previous two decades. It refers to the manifold process, which comprises of doing observations, raising the questions, enquiring the books and relevant information sources to determine what is already present in the body of knowledge, planning the research through several tools, analyzing, interpreting, and generalizing the data with the assistance of explanations and descriptions (Ulfah, 2018). The definitional framework revolves around the observations and derives results from the particular data. The literature indicates that students learning is enhanced while studying in the scientific classrooms (Lai et al., 2018). Some of the studies also have contrary results. Mason et al. (2013) articulated no fluctuations between the results of the students who attend lectures in the scientific environment and those who have been instructed chemistry in a conventional manner. Conversely, Leonard et al. (2001) noted that students have grasped the concepts efficiently in the inquiry-based learning of biology and achieved higher scores comparatively. Furthermore, the existing literature has shown that scientific inquiry enhances the students’ retention.

Self-efficacy has been narrated as an individual perception of their abilities to execute an action successfully. It is not the concrete criteria to measure abilities, rather it is a perception of the capability to perform the tasks (Sekerdej and Szwed, 2021). Self-efficacy can influence individual behavior and regulate choices. As far as the association between self-efficacy and learning is concerned, higher self-efficacy has been depicted as inclined to preserve in the critical period (Hung et al., 2020). It also engages students and helps forge specific mechanisms to improve the learning process. However, those students who posit lower self-efficacy are prone to poor performance. Eventually, they perceive failure as an outcome of fragile talents and bad luck. Nonetheless, self-efficacy can mediate attitude but exerts stress over the output. The immense level of self-efficacy predicts students’ interests in sciences and their career. Tai et al. (2006) put forward that convincing students about their abilities to deal with science has been a key factor for students to enroll in sciences in their upcoming years. The pattern of failure has been altered with the view that science is achievable. Perhaps, academic outcomes and performance can be enhanced by embedding strong self-efficacy. Science inquiry self-efficacy has been put forward as a moderator in the present model. Scientific inquiry-based self-efficacy has been associated with graphical attraction because it improves students’ academic performance. Scientific inquiry emanates from the BSG when students design rounds through animation and 3D models. Network support encourages scientific inquiry to comprehend the different physical laws (Chang et al., 2019). Thus, the following hypothesis has been made.

H6: Simulation games’ self-efficacy positively moderates the relationship between the perceived TTF and online learners’ intentions to use BSG.

## Materials and Methods

This study used structural equation modeling to examine the proposed model because the study includes several exogenous factors with multidimensional interactions. The following subsection discussed instrument development and the data collection process, followed by the descriptive profile of the collected response set.

### Instrument

To avoid instrumental reliability issues, the authors adapted the items for each construct from existing sources and literature. Furthermore, the instrument was adopted in the context of the current research. Particularly, the four items scale to examine real-time interactivity and inquiry-based simulation games’ self-efficacy adapted from the research contribution by Nietfeld et al. (2006) and Etemad-sajadi (2016), respectively. Moreover, the three-item scale was adapted from the work by Hsu et al. (2012); Zhou et al. (2010), and Kim and Jae (2019) to map graphical attraction, TTF, and flow experience, respectively. In addition, the intentions to use business simulation games and active control are underlined by using the three-item scale researched by Zulfiqar et al. (2017) and Alkali and Mansor (2017), respectively. All the adapted items for this study mapped over the continuum of a five-point Likert scale, where 1 is strongly disagree and 5 is strongly agree. The details of the adapted instrument are listed in Table 1.

**TABLE 1:** Adapted instrument with listed sources.

Construct	Items	Source
Graphical attraction (GA)	(1). The business simulation environment’s graphics are eye-catching. (2). The business simulation environment’s graphics are creative. (3). The business simulation environment’s graphics are visually appealing.	Hsu et al., 2012
Real-time interactivity (RI)	(1). The business simulation environment allows me to interact with it to receive information in a virtual business environment. (2). The business simulation environment has interactive features to help me understand business activities. (3). The business simulation environment allows me to find the desired information easily without involving my instructor/tutor. (4). The interaction with the business simulation environment is efficient.	Etemad-sajadi, 2016
Flow experience (FE)	(1). The interaction using a business simulation environment is interesting. (2). When using the business simulation environment, I feel the excitement of exploring. (3). I am absorbed when using a business simulation environment.	Kim and Jae, 2019
Active control (AC)	(1). I feel I have a lot of control over my use of the business simulation environment. (2). While using the business simulation environment, I should choose freely what I want to see. (3). When using the business simulation environment, my actions should decide the kind of experience I get.	Alkali and Mansor, 2017
Task technology fit (TTF)	(1). The business simulation functions are sufficient in helping me to complete the business management course learning. (2). The business simulation functions are appropriate in helping me to complete the business management course learning. (3). In general, the functions of a business simulation game help me to understand my business course learning.	Zhou et al., 2010
Simulation games self-efficacy (in a scientific manner) (SGE)	(1). I am sure that I can learn in business in a simulation environment. (2). I can get a good grade in the related academic course using a business simulation environment. (3). I have a lot of self-confidence when it comes to learning business studies in a business simulation environment. (4). Even before I begin this business course in the semester, I feel confident I’ll be able to understand the business simulation environment.	Nietfeld et al., 2006
Intentions to use business simulation game (IUG)	(1). I intend to use a business simulation environment. (2). I would like to use a business simulation environment. (3). If my friends or class fellows looked for any guide to improving their learning, I would recommend a business simulation environment.	Zulfiqar et al., 2017

### Data Collection

To address the hygiene concerns and safety precautions during coronavirus disease 2019 (COVID-19), the authors used the online questionnaire survey method to collect the survey. Particularly, the convenience sampling method was adopted, where students attending a business simulation course (at undergraduate and postgraduate level) *via* online classes were approached. The researchers collected samples from the affiliated universities and through referral networks only. The authors assumed that the response would be taken anonymously to keep the data confidential and address privacy concerns. The data were collected during the first and second quarters of 2020 from mainland China. A total of 523 complete responses were collected, whereas the descriptions of the collected sample are given in Table 2 (further discussed in Section 6). The responses of the earlier and later half were compared and showed no difference between the collected subsets. Thus, the responses eliminated the risk of non-response bias.

**TABLE 2:** Descriptive of the collected sample.

Characteristic	Detail	Freq.	In Percentage
Gender	Male Female	317 206	60.61 39.39
Education	Diploma (course only) Undergraduate Postgraduate	35 313 175	06.69 59.85 33.46
Major (discipline)	Business Studies Management and Engineering Others	261 242 20	49.91 46.27 03.82

*Freq. = Frequency.*

## Analysis

SPSS-Statistics (version 26) and AMOS (version 24) were employed to examine the proposed model of the research. The analysis comprised of (1) data normality and common method bias testing, (2) the constructs’ internal and external validity and reliability testing by examining confirmatory factor analysis, (3) structural path analysis, and (4) moderation effect as proposed in Section 3.

### Data Normality Testing

Data skewness measured by a kurtosis marker was calculated to examine data normality and biases in the collected data. Multicollinearity (variance inflation factor) was checked in the skewness measure. All the markers and checks were counted under the upper limit, as Hair et al. (2014) advised. The single method biases were also calculated as a single method adopted by the authors for data collection. The maximum variance was calculated as 43.21%. Therefore, the study is free of data biases risk because Harman’s single factor analysis concluded satisfactory results, as recommended by Podsakoff et al. (2003).

### Measurement Estimation

The confirmatory factor analysis was performed to compute the external and internal validity of the data. The factor loadings of the surveyed data at a saturated and model level were calculated. Furthermore, Cronbach alpha was also calculated to check data reliability. Satisfactory results are noted, as shown in Table 3. All the value mentioned earlier is above the recommended lower acceptable limit of.700, as advised by Hair et al. (2014).

**TABLE 3:** Factor loadings and the Cronbach’s alpha values.

Construct	Items	Loadings (CFA)	Loadings (Model)	Alpha
Real-time interactivity (RI)	RI1 RI2 RI3 RI4	0.980 0.970 0.920 0.930	0.980 0.970 0.920 0.930	0.974
Simulation games self-efficacy (SGE)	SGE1 SGE2 SGE3 SGE4	0.970 0.930 0.850 0.880	0.970 0.930 0.850 0.880	0.947
Task-technology-fit (TTF)	TF1 TF2 TF3	0.980 0.970 0.960	0.980 0.970 0.960	0.979
Graphical attraction (GA)	GA1 GA2 GA3	0.950 0.890 0.820	0.950 0.890 0.830	0.917
Intentions to use business simulation game (IUG)	IUG1 IUG2 IUG3	0.940 0.900 0.890	0.940 0.890 0.890	0.933
Flow eperience (FE)	FE1 FE2 FE3	0.960 0.910 0.880	0.960 0.910 0.880	0.941
Active control (AC)	AC1 AC2 AC3	0.920 0.790 0.870	0.910 0.790 0.870	0.894

*Alpha, Cronbach Alpha; CFA, Confirmatory Factor Analysis; Loadings, Factor Loadings.*

Apart from factor loadings and Cronbach alpha-based analysis, average variance, composite reliability, the interaction effect of average variance extracted with interconstruct correlation, and hetro/mono trait are also measures, as shown in Table 4. The literature argues that the composite reliability’s lowest acceptable value is 0.700 (Hair et al., 2014), the average variance extracted must not be below.500 (Pallant, 2016), the interaction between interconstruct correlation must not bypass the scores of the square root of relative average variance extracted (Fornell and Larcker, 1981), and hetromono traits scores must not be above.800 (Henseler, 2017). All the acceptable scores are reported in Table 4. The model fit indices were also computed on the saturated level, where the acceptable lower cut-offs were recorded, as listed in Table 5.

**TABLE 4:** Composite reliability, average variance extracted, and heterotrait-monotrait testing.

	CR	AVE	RI	SGE	TF	GA	IUG	FE	AC
Real-time Interactivity (RI)	0.974	0.903	0.950	0.495	0.400	0.235	0.359	0.473	0.394
Simulation Games Self-Efficacy (SGE)	0.948	0.822	**0.493**	0.906	0.564	0.491	0.605	0.669	0.645
Task-Technology-Fit (TTF)	0.979	0.940	**0.404**	**0.544**	0.969	0.340	0.457	0.474	0.442
Graphical Attraction (GA)	0.919	0.792	**0.227**	**0.471**	**0.346**	0.890	0.440	0.420	0.437
Intentions to Use Buss Sim Game (IUG)	0.934	0.825	**0.354**	**0.581**	**0.458**	**0.450**	0.908	0.531	0.585
Flow Experience (FE)	0.942	0.845	**0.471**	**0.655**	**0.458**	**0.421**	**0.517**	0.919	0.530
Active Control (AC)	0.896	0.742	**0.394**	**0.633**	**0.422**	**0.431**	**0.576**	**0.517**	0.861

*Numbers in italics are hetro/mono trait results, underlined numbers are the square root of relative average variance extracted, and the numbers in bold are correlation scores. CR, Composite reliability; AVE, Average Variance Extracted.*

**TABLE 5:** Model fit indices.

Measures	Threshold	Estimated	Model
CMIN	-	566.071	760.163
Df	-	209	215
CMIN/df	Less than 5.0	2.708	3.536
CFI	Above 0.950	0.975	0.962
GFI	Above 0.950	0.947	0.930
TLI	Above 0.950	0.970	0.955
NFI	Above 0.950	0.961	0.947
RMSEA	Less than 0.080	0.057	0.070

*CMIN, Chi-square; df, degree of freedom; CFI, comparative fit index; GFI, goodness of fit index; TLI, Tucker Lewis Index; NFI, Normed Fit Index; RMSEA, root mean square error of approximation.*

Given that the study comprises adapted instruments, the authors adopted Harmans’ single factor analysis to examine the risk of common method bias, as suggested by Podsakoff et al. (2003). Specifically, the variance recorded is 26.74%, which is below 50%. Thus, the study eliminated the risk of common method bias. Furthermore, the common latent factor analysis was performed following Keren et al. (2021). The regressions (standardized) of the model with common latent factors and without latent factors were compared. The authors observed no difference between their standardized regression weights above.200. Thus, the study excluded the risk of common method bias.

### Model Hypotheses and Moderation Testing

The model fit indices for the structured model were computed. Particularly, the model fit measured the discrepancy between sigma and unrestricted sigma (CMIN), population discrepancy (by measuring RMSEA), comparing baseline model (by computing TLI and NFI), and the goodness-of-fit (GFI). The acceptable score was reported as advised by Hu and Bentler (1999). The estimated and proposed model is shown in Table 5.

**FIGURE 1:**
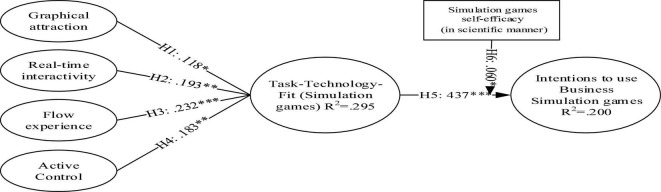
Pictorial view of the proposed model. ***p < 0.001, **p < 0.01, *p < 0.05.

Furthermore, all the proposed hypotheses in Section 3 (H1-H5) recorded significance. Each of the proposed hypotheses is discussed in Section 6. Furthermore, the variance explained by the perceived TTF and by the perceived intentions to use business simulation games is.295 and.200, respectively. The pictorial view of the structural path analysis is shown in Figure 1.

**FIGURE 2:**
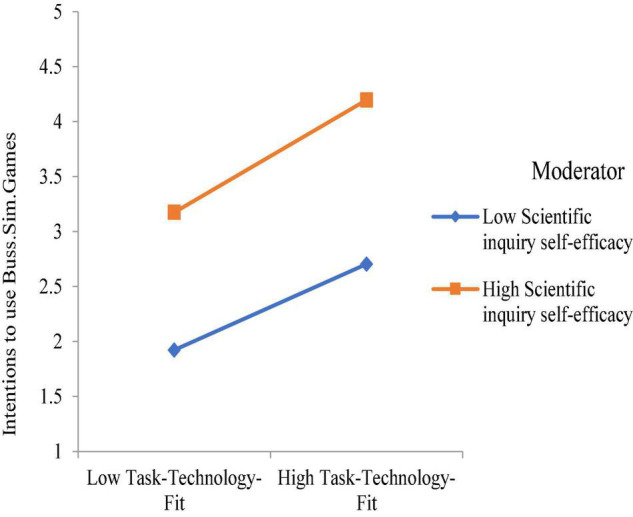
Interaction plot for the moderating effect of the perceived simulation games self-efficacy over the relationship between the perceived TTF and the intention to use BSG.

The SPSS process macro (model 1) was used, where the bootstrapping method was adopted, to measure the moderation effect of the simulation games self-efficacy over the relationship between the perceived TTF and the perceived intentions to use BSG. The findings concluded that the perceived simulation games self-efficacy, which is fundamentally designed based on individuals’ scientific inquiry, strengthened the positive relationship between the learners’ perceived TTF, and intention to use BSG. The graphical interaction plot of the proposed moderator is shown in Figure 2.

## Discussion and Implication

This study has been conducted in the context of the students’ intention to use BSG to increase their academic performance. Regarding the demographic characteristics, particularly the gender distribution, males constituted 60.61% of the total participants, and females were 39.39%. Regarding education, 6.69% possess a diploma, 59.85% are undergraduate, and 33.46% are postgraduate. Concerning disciplines, BSG constituted 49.91% of the total participants, management had 46.27%, and engineering had 3.82%. However, the study put forward that simulation games’ self-efficacy has been identified as a scientific inquiry that fosters a stronger association between TTF and the intention to use BSG.

This study adopted TTF to investigate the factors affecting students’ academic performance *via* immersive media learning, which remarked interesting results through findings that can be bifurcated on the basis of underlined research questions of the study in the first segment discussed later. While addressing and exploring RQ1, that is, how interactivity and flow experience help define students’ perception toward TTF, the study articulated a surprising fact that flow experience possesses the strongest exogenous compared with the rest of the factors (graphical attraction and real-time interactivity) to define the perceived TTF among learners in the case of BSG adoption (H3: β = 0.232, ρ ≤ 0.001). It is followed by the role of interactivity, which includes active control and real-time interactivity (H4: β = 0.183, ρ ≤ 0.01) and (H2: β = 0.193, ρ ≤ 0.01). In addition, the richness appears as a fragile factor to predict the mapped-out model. Its least significance in the construct can be depicted by H2 (β = 0.118, ρ ≤ 0.05) while determining the perceived TTF among learners in the case of BSG adoption. These findings have contributed in a manner that can be observed in existing literature (Estrada et al., 2019; Al-rahmi et al., 2020; Bitrián et al., 2020; Bolotaolo et al., 2021). Furthermore, perceived TTF among learners is noted as the most significant and strongest determinant while defining the learners’ intention to use BSG (IUG) at the higher education level (H2: β = 0.437, ρ ≤ 0.001). The findings can be stated in-line with the literature. At last, RQ2, that is, how the scientific (inquiry based) self-efficacy was tested as moderator while examining the association between TTF and intentions to use BSG for learning purposes, was considered. The findings put forward that the role of scientific (inquiry based) self-efficacy strengthens the association between the measured TTF and intentions to use BSG for learning purposes (H2: β = 0.060, ρ ≤ 0.05).

This study entails diverse theoretical implications. First, the study has put forward flow and quality in terms of the information and interfaces that have been considered within the context of TTF in the case of game simulation (Mosiane and Brown, 2020), which can be a novel contribution to the literature. The role of interactivity has been scarcely discussed in the literature because it has not been investigated much in the academic sphere. Second, the study has come up with a unique contribution that determines the role of scientific inquiry-based self-efficacy to underline its constructive role in defining the positive intentions of learners to use a business simulation environment. Third, the study implies that, in gamification, TTF, the essence addressing design-based attributes, user experience, and behavior, should be addressed. This study can be captioned as a unique theoretical contribution in the case of the higher education context because the study includes each of the afore discussed concepts of gamification in this research.

The practical implications of the study have significance at the policy-making level. The collected survey has been conducted during COVID-19 pandemic. Thus, stating that simulation game-based learning in the time of COVID-19 has become more challenging is important because task-related characteristics can be more critical determinants in recent time. Furthermore, the study concludes that, although flow experience is a significant determinant, the technical characteristics, that is, graphical attraction, are less effective in shaping learners’ intentions to adopt BSG. It implies that the task attributes in online gaming dampen the positivity of technical characteristics, which needs to be researched further. Moreover, in the diverse spectrum of implications for educators, efforts to minimize the psychological gap learners used to experience because of the online teaching environment during the pandemic is essential.

The study’s practical significance can be elucidated in terms of the exposures because TTF enhances the user’s excitement in the context of the BSG and the learning outcome is not at the same pace. In the presence of flow experience, the real-time interactivity and active control have enhanced playfulness and learning at the same continuum. Nevertheless, the scientific self-efficacy will rise in this context of BSG. In addition, for BSG providers, technology support needs a self-assistive environment and tools, which can help learners to practice BSG in the extended mixed approach, where online and offline BSG usage can help learners to maximize their learning outcomes and objectives. Findings show that the scientific self-efficacy (inquiry-based) characteristics of learners’ cognition can help to improve learning through BSG for learners. It concludes that self-efficacy to inquire and digitally seek information can be critical in formulating learners’ self-regulated learning. Furthermore, the domination of flow experience also highlights the potential risk where flow experience can be accounted for limited learning outcomes and leads to cognitive-behavioral outcomes (i.e., games addiction). The level of effort educators is giving in online BSG-based learning needs to be examined qualitatively. It can underline the challenges educators experience because of the virtual learning environment. Future studies can examine how task characteristics (in the view of TTF) can affect technology characteristics concerning BSG.

## Conclusion and Future Studies

The present model of the study entails several limitations that indicate the need to bridge these academic gaps in the context of intentions to use BSG. This study is survey-based, which is a limitation. Future studies can find basis from an experimental research design to determine the intentions to use BSG. Future studies can have qualitative examinations of this particular phenomenon. Similarly, flow experience can be enquired with reference to the human-computer interaction, which can yield novel contributions. At last, the readiness of the technology has been studied as the moderator in the underlined model. It can be further studied as the exogenous factor to dig out its impact on the students or the general population. The current venture encompasses the interesting outcomes that indicate the learners’ intentions to adopt the business simulation games. Our study concludes that flow experience has been the most influential factor to determine the use of business simulation games. On the other hand, media richness has been a low-affective factor to measure the concerned model. However, the scientific efficacy has been essential to shaping individual behavior for enhanced learning through BSG.

In the recent pandemic, the significant role of BSG has increased. Nevertheless, technological advancement has motivated learners to engage and learn through the games. The present construct concluded that, to use BSG, learners have a significant role in the flow experience that derives their behavior more than any other factor. The rationale may be embedded in the motivation and the engagement that learners seek during engagement with these games. Thus, BSG is evolving as an alternative mode of learning owing to its interesting features.

## Data Availability Statement

The raw data supporting the conclusions of this article will be made available by the authors, without undue reservation.

## Ethics Statement

The studies involving human participants were reviewed and approved by the Ethical Committee of the Affiliated University (School of Public Affairs, University of Science and Technology of China). The patients/participants provided their written informed consent to participate in this study.

## Author Contributions

HH initially drafted the manuscript. YW significantly contributed to each section of the manuscript at the time of revision. Both authors contributed to the article and approved the submitted version.

## Conflict of Interest

The authors declare that the research was conducted in the absence of any commercial or financial relationships that could be construed as a potential conflict of interest.

## Publisher’s Note

All claims expressed in this article are solely those of the authors and do not necessarily represent those of their affiliated organizations, or those of the publisher, the editors and the reviewers. Any product that may be evaluated in this article, or claim that may be made by its manufacturer, is not guaranteed or endorsed by the publisher.
